# Application of artificial intelligence in differentiating IgG4-related ophthalmic disease and orbital MALT lymphoma: a review of radiomics and deep learning advances

**DOI:** 10.3389/fimmu.2026.1722733

**Published:** 2026-02-11

**Authors:** Wei Weng, Yaomeng Chen, Rouhui Jin, Jinchun Chen, Yuantong Gao, Yongchou Li, Haochen Shao, Shitian Bao, Weikuan Xue

**Affiliations:** 1 Department of Radiology, The Third Affiliated Hospital of Wenzhou Medical University, Wenzhou, Zhejiang, China; 2 Department of Radiology, Wenzhou People’s Hospital, Wenzhou, Zhejiang, China; 3 Department of Pathology, The Third Affiliated Hospital of Wenzhou Medical University, Wenzhou, Zhejiang, China

**Keywords:** artificial intelligence, deep learning, differential diagnosis, IgG4-related ophthalmic disease, orbital lymphoma, orbital MALT lymphoma, radiomics

## Abstract

The differentiation between Immunoglobulin G4-related ophthalmic disease (IgG4-ROD) and orbital lymphoma, particularly the mucosa-associated lymphoid tissue (MALT) subtype, presents a significant clinical challenge due to overlapping imaging features and similar presentations. Recent advances in artificial intelligence (AI), particularly radiomics and deep learning, have shown promising potential in enhancing diagnostic accuracy by extracting high-dimensional imaging features and constructing robust predictive models. This review systematically examines the current state of AI applications in distinguishing IgG4-ROD from orbital MALT lymphoma, highlighting key methodologies in image-based feature extraction, model development, and diagnostic performance evaluation. We explore various AI techniques applied to multimodal imaging data integration and discuss optimization strategies for deep learning architectures tailored to this clinical context. Additionally, the review addresses the practical challenges and limitations of translating AI-assisted diagnostic tools into routine clinical practice, including issues related to small sample sizes, retrospective single-center designs, data variability, interpretability, and the critical need for robust external validation. By synthesizing recent research findings, this review aims to provide a comprehensive overview of AI-driven diagnostic advances, critically assess current challenges, and propose future directions to improve the accuracy and reliability of orbital disease differentiation, ultimately supporting more precise clinical decision-making.

## 1 Introduction

Immunoglobulin G4-related ophthalmic disease (IgG4-ROD) and orbital mucosa-associated lymphoid tissue (MALT) lymphoma represent two predominant pathological entities affecting the orbital region (1, 2). Both conditions frequently manifest with overlapping clinical symptoms and share remarkably similar conventional imaging features on computed tomography (CT) and magnetic resonance imaging (MRI), which poses a significant challenge for accurate differential diagnosis in clinical practice.

IgG4-ROD is a chronic immune-mediated fibroinflammatory disorder histopathologically characterized by dense infiltration of IgG4-positive plasma cells, storiform fibrosis, and obliterative phlebitis within affected orbital tissues (3–5). Clinically, it commonly presents as eyelid swelling, proptosis, diplopia, and visual impairment, often involving the lacrimal glands, extraocular muscles, orbital soft tissues, and occasionally the trigeminal nerves. The disease frequently presents with bilateral involvement, with lacrimal gland enlargement (often spindle or kidney-shaped with well-defined margins) being a prevalent manifestation (3, 4, 6). On T_2_-weighted images (T_2_WI), lesions typically display low to iso-intensity signals with homogeneous or delayed contrast enhancement. Infraorbital nerve enlargement is a characteristic imaging feature, reflecting perineural involvement (7). While serum IgG4 levels are typically elevated, this finding is not exclusively diagnostic (3, 4, 8–11).

Conversely, orbital lymphoma, predominantly extranodal marginal zone B-cell lymphoma of MALT (MALT lymphoma), is the most common malignant orbital tumor and a significant subset of orbital lymphoproliferative disorders (8, 12). Pathologically, MALT lymphoma arises from monoclonal lymphoid proliferation of marginal zone B-cells, and recent studies have also implicated Chlamydia psittaci infection in its pathogenesis (13, 14). Clinically, it typically presents as a painless, slowly enlarging orbital mass causing proptosis, eyelid swelling, and sometimes diplopia, commonly affecting older adults (15, 16). Imaging features on MRI and CT often show well-defined soft tissue masses, frequently unilateral, with isointense signals on T_1_-weighted images (T_1_WI) and hyperintense signals on T_2_WI. Contrast enhancement is usually heterogeneous (8, 12). Unlike IgG4-ROD, orbital lymphoma may demonstrate irregular lesion morphology and can be associated with bony erosion in some high-grade lymphomas, though less common in MALT lymphoma. Infraorbital nerve enlargement can also be present but is less specific (7). The diagnostic conundrum is further heightened by the rare instances where IgG4-ROD and MALT lymphoma can coexist or even transform into each other, further complicating the clinical picture (15, 17). Therefore, this review primarily focuses on the differentiation between IgG4-ROD and orbital MALT lymphoma, as it represents the most common subtype and the greatest diagnostic challenge due to its indolent nature and strikingly similar clinical and radiological presentations (18) (**Table 1**). However, the broader term ‘orbital lymphoma’ is used in this text when referring to general screening principles or studies that included mixed histological subtypes.

**Table 1:** Comparison of clinical and imaging features between IgG4-related ophthalmic disease (IgG4-ROD) and orbital MALT lymphoma.

Feature	Location of involvement	T1WI signal	T2WI Signal	Enhancement pattern	Margins	Lymphadenopathy	Nerve involvement	Bilateral involvement	Serum IgG4 levels	Pathological features
IgG4-ROD	Mainly involves orbital soft tissues, lacrimal glands, extraocular muscles, and trigeminal nerves	Low to iso-intensity	Low to iso-intensity	Homogeneous or delayed enhancement	Usually well-defined, spindle or kidney-shaped	Not typically involved or mildly enlarged	Common involvement of the trigeminal nerve, may extend to adjacent foramina	Commonly bilateral, especially in lacrimal glands	Typically elevated	IgG4-positive plasma cell infiltration, storiform fibrosis, obliterative phlebitis
Orbital MALT Lymphoma	Primarily affects orbital soft tissues, lacrimal glands, and conjunctiva	Iso-intensity or slightly low	High intensity	Variable, often homogeneous or mildly heterogeneous	Variable, typically well-defined margins, but irregular in aggressive lesions.	Regional lymph node involvement is uncommon; if present, it suggests potential dissemination.	Nerve involvement can occur, but it is less characteristic or prevalent than in IgG4-ROD.	Typically unilateral, rarely bilateral	Normal IgG4 levels	Monoclonal B-cell proliferation, tumor cells typically small or medium-sized B-cells

IgG4, Immunoglobulin G4; IgG4-ROD, Immunoglobulin G4-Related Ophthalmic Disease; Orbital MALT Lymphoma, Orbital Mucosa-Associated Lymphoid Tissue Lymphoma; T1WI, T1-weighted image; T2WI, T2-weighted image.

The gold standard for definitive diagnosis traditionally relies on invasive tissue biopsy followed by histopathological and immunohistochemical analyses. While biopsy provides crucial information on cellular morphology, clonality, and protein expression, it is an invasive procedure associated with patient discomfort, procedural risks, and potential sampling errors, particularly given the anatomical complexity of the orbit (19). Moreover, biopsy results may require extended processing time, delaying therapeutic decision-making. Despite characteristic clinical and imaging features, distinguishing IgG4-ROD from orbital lymphomas, particularly MALT lymphoma, remains challenging due to their overlapping presentations and imaging heterogeneity (18). Both conditions frequently manifest as orbital soft tissue masses involving the lacrimal gland and extraocular muscles, with similar enhancement patterns on CT and MRI. For instance, while IgG4-ROD lesions often show low to iso-intensity on T_2_WI and lymphomas tend to be hyperintense, exceptions and overlaps exist, especially in early or atypical cases. Additionally, both diseases may present with infraorbital nerve enlargement and bilateral involvement, complicating differentiation (7). Morphological imaging signs such as lesion shape, boundary definition, and enhancement heterogeneity have limited specificity. Conventional imaging modalities rely heavily on radiologist experience and subjective interpretation, contributing to diagnostic variability and potential misdiagnosis, which can lead to inappropriate treatment. Moreover, the presence of IgG4-positive plasma cell infiltration within lymphoma tissue in some cases can lead to IgG4-ROD mimicking lymphoma, further blurring distinctions (15, 20). This diagnostic ambiguity underscores the unmet need for objective, high-precision, non-invasive imaging biomarkers and analytical techniques.

In recent years, artificial intelligence (AI) has emerged as a transformative technology in medical imaging analysis, offering new possibilities to overcome diagnostic challenges in complex diseases. While the broader spectrum of AI in medicine includes diverse techniques such as natural language processing and unsupervised clustering, the field of orbital differentiation is predominantly driven by radiomics and deep learning. These two methodologies are particularly pertinent for this specific clinical task because radiomics excels at quantifying subtle intratumoral heterogeneity through texture analysis, while deep learning is uniquely capable of automatically learning complex, hierarchical spatial patterns essential for characterizing soft tissue lesions. AI encompasses a range of computational methods, including machine learning, deep learning, and radiomics, which enable the extraction and interpretation of high-dimensional quantitative features from medical images beyond human visual perception (21, 22). Radiomics involves systematic extraction of handcrafted imaging features such as texture, shape, and intensity, while deep learning utilizes neural networks to automatically learn hierarchical image representations. These AI-driven approaches have demonstrated remarkable potential in improving disease detection, characterization, and prognostication across various medical fields, including oncology, neurology, and cardiovascular imaging (23, 24).

Specifically, in the context of orbital diseases, AI techniques have begun to be applied to differentiate IgG4-ROD from orbital MALT lymphoma by analyzing imaging modalities such as CT, MRI, and histopathological slides. For instance, radiomics models based on MRI have shown promising results in capturing subtle image features that discriminate between these entities, achieving higher diagnostic accuracy compared to conventional radiological assessment (8, 25). Deep learning models trained on hematoxylin-eosin (HE) stained pathological images have also demonstrated superior performance in distinguishing IgG4-ROD from MALT lymphoma, even outperforming expert ophthalmologists in some studies (26). Furthermore, the integration of multi-regional radiomic features from multiparametric MRI using advanced machine learning classifiers has shown potential to outperform subspecialty radiologists in differentiating these diseases, highlighting the capability of AI to augment clinical decision-making (27). Beyond imaging, molecular and genetic analyses combined with AI-based data mining have identified distinct gene expression profiles and microRNA signatures that further aid in understanding the pathogenesis and improving diagnostic precision (1, 2, 28, 29).

Despite these advances, challenges remain in standardizing AI methodologies, ensuring robust external validation, and integrating AI tools into routine clinical workflows. Current studies often involve limited sample sizes and lack prospective multicenter validations, underscoring the need for larger, well-designed investigations to confirm the clinical utility of AI-assisted diagnostics in orbital lymphoproliferative diseases (25, 30). Moreover, the interpretability of AI models and their acceptance by clinicians are critical factors for successful translation into practice (21). Addressing these issues will pave the way for AI-driven precision medicine approaches that can improve patient outcomes by enabling early, accurate, and non-invasive differentiation between IgG4-ROD and orbital MALT lymphoma.

This review aims to systematically summarize the current landscape of AI applications, focusing on radiomics and deep learning techniques, in the differential diagnosis of IgG4-related ophthalmic disease and orbital MALT lymphoma. We will critically analyze the strengths and limitations of existing studies, discuss the technological and clinical challenges, and explore future directions for research and clinical translation in this rapidly evolving field. This review is structured as a narrative review, aiming to provide a comprehensive overview and critical synthesis of the current advancements, challenges, and future directions in this specific area. To achieve this, a focused literature search was conducted primarily using Google Scholar. The search strategy involved specific keyword combinations to identify relevant literature. These combinations included: “orbital lymphoma” AND IgG4-ROD, “orbital MALT lymphoma” AND IgG4-ROD, and further refined searches by adding AI methodologies such as “radiomics” or “Deep Learning” (e.g., “orbital lymphoma” AND IgG4-ROD AND radiomics, “orbital MALT lymphoma” AND IgG4-ROD AND “Deep Learning”). Emphasis was placed on identifying highly relevant, impactful, and recent studies that address the application of AI in the differential diagnosis of these conditions, ensuring a broad coverage of the field’s progress. Distinct from general overviews of AI in ophthalmology, this review uniquely synthesizes the specific methodological nuances required to distinguish these two clinically mimicking entities. By critically dissecting the translational hurdles—ranging from small-sample constraints to model interpretability, we aim to provide a concrete framework that guides future research toward clinical implementation.

## 2 AI-driven approaches for differentiating IgG4-ROD and orbital MALT lymphoma

**Figure 1:**
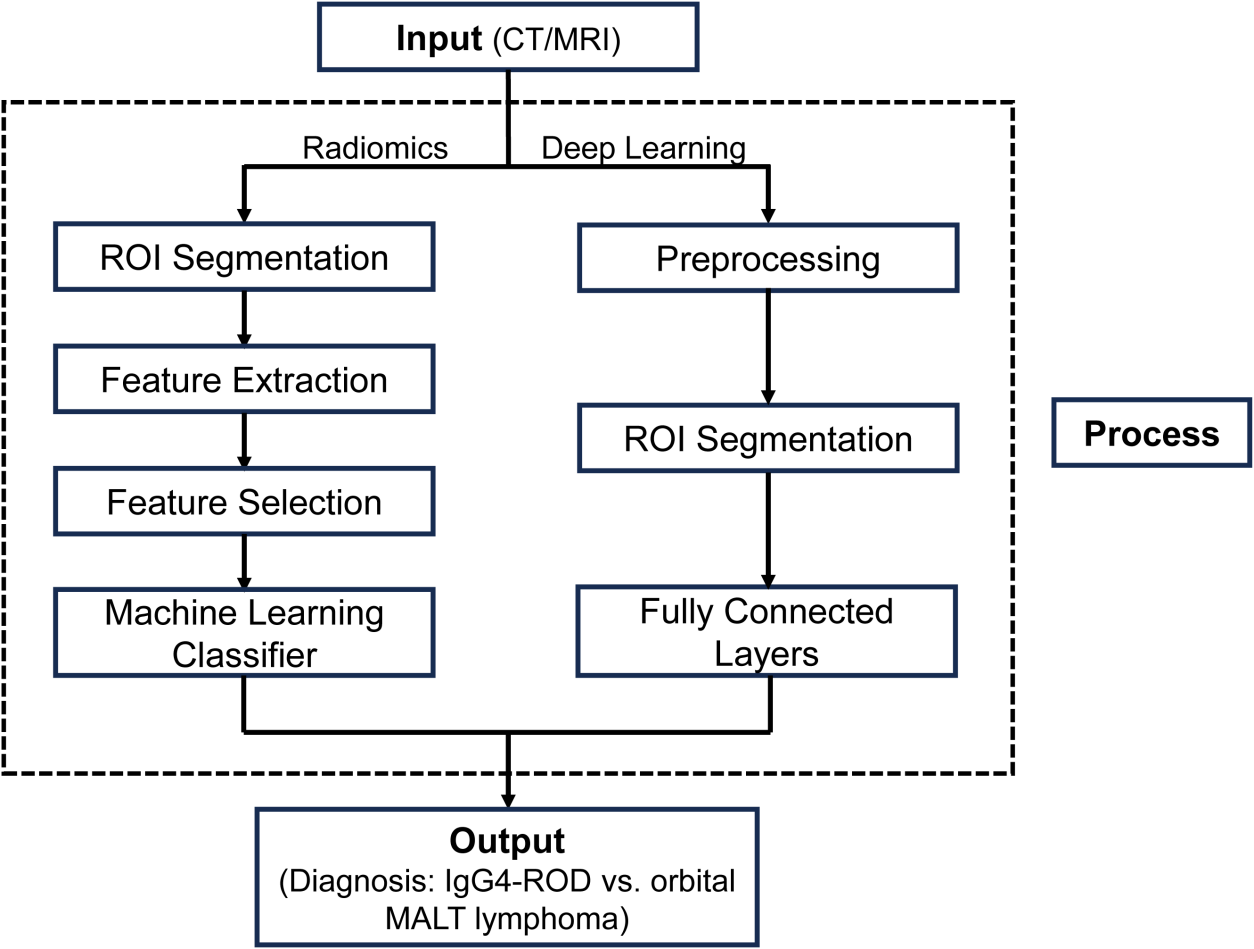
Schematic representation of the typical Artificial Intelligence (AI) workflow for differentiating orbital diseases. The pipeline begins with the acquisition of medical images (CT or MRI). Radiomics Approach: Involves segmentation of the Region of Interest (ROI), extraction of high-dimensional handcrafted features (morphology, texture, intensity), feature selection to reduce dimensionality, and classification using machine learning algorithms. Deep Learning Approach: Utilizes convolutional neural networks (CNNs) to automatically learn hierarchical feature representations directly from the raw images or ROIs for prediction. Both pathways aim to output a probability score for the differential diagnosis between IgG4-ROD and orbital MALT lymphoma. CT, Computed Tomography; IgG4-ROD, Immunoglobulin G4-Related Ophthalmic Disease; MALT Lymphoma, Mucosa-Associated Lymphoid Tissue Lymphoma; MRI, Magnetic Resonance Imaging; ROI, Region of Interest.

The inherent limitations of conventional imaging in reliably distinguishing IgG4-ROD from orbital MALT lymphoma, due to their overlapping features and imaging heterogeneity, have created a significant unmet need for objective and high-precision diagnostic tools (18). AI techniques, particularly radiomics and deep learning, offer promising avenues to address this diagnostic challenge by extracting quantitative imaging features and learning complex patterns beyond human visual assessment. A schematic representation of the typical workflows for these two methodologies is illustrated in **Figure 1**. This section will delve into the advances and challenges of these AI methodologies in improving the differential diagnosis of these two orbital conditions.

### 2.1 Radiomics in differentiating IgG4-ROD and orbital lymphoma

#### 2.1.1 Overview of radiomics technology, workflow, and advantages

Radiomics is an advanced quantitative imaging analysis technique that extracts a large number of features from standard medical images, providing a high-throughput approach to characterize tissue heterogeneity beyond what is visually appreciable (21, 22). These features encompass multiple dimensions, including morphological parameters (such as volume and shape irregularity), first-order intensity statistics (e.g., pixel value distributions), texture features derived from gray-level co-occurrence matrices (GLCM) and gray-level run-length matrices (GLRLM), and higher-order features obtained through transformations like wavelet decomposition. The typical radiomics workflow begins with image acquisition, followed by manual or semi-automated segmentation of regions of interest (ROIs) that delineate the lesion or tissue under study. Subsequently, a comprehensive set of quantitative features is extracted from these defined ROIs (31). Due to the high dimensionality and potential redundancy of features, feature selection and dimensionality reduction techniques such as Least Absolute Shrinkage and Selection Operator (LASSO) regression or Principal Component Analysis (PCA) are employed to identify the most informative and robust features. Finally, predictive or classification models are constructed using machine learning algorithms and validated through internal cross-validation or external datasets to ensure generalizability. Radiomics offers several advantages in differentiating IgG4-ROD from orbital MALT lymphomas, which often present with overlapping clinical and imaging characteristics. By capturing subtle imaging heterogeneity and microstructural differences invisible to the naked eye, radiomics can improve diagnostic accuracy, reduce reliance on invasive biopsy procedures, and potentially provide prognostic information. This quantitative approach complements conventional imaging interpretation and may facilitate personalized treatment strategies in orbital diseases (8, 25, 27).

#### 2.1.2 Radiomics feature extraction and machine learning model construction

In studies aimed at distinguishing IgG4-ROD from orbital MALT lymphomas, radiomics feature extraction typically involves first-order statistics (mean, variance, skewness), second-order texture features derived from GLCM (contrast, correlation, homogeneity) and GLRLM (short-run emphasis, long-run emphasis), morphological descriptors (shape, compactness), and advanced features from wavelet or other filter-based transformations (8, 25). These features quantify lesion heterogeneity, texture complexity, and shape irregularities that may reflect underlying pathological differences. Given the high dimensionality of extracted features, robust feature selection methods such as LASSO regression and PCA are crucial to reduce overfitting and enhance model interpretability by isolating the most discriminative features. Subsequently, various machine learning classifiers are employed, each offering distinct advantages regarding performance and explainability. Linear models, such as Logistic Regression (LR) and Support Vector Machines (SVM), are widely used due to their simplicity and high interpretability; the weights assigned to each feature are transparent, allowing clinicians to understand which imaging characteristics (e.g., texture *vs*. shape) drive the diagnosis. However, their capacity to model complex, non-linear relationships is limited. In contrast, ensemble methods like Random Forests (RF) and XGBoost typically achieve higher diagnostic performance (accuracy and AUC) by capturing non-linear interactions between features. The trade-off is reduced explainability; while “feature importance” rankings can be visualized, the specific decision-making process for individual patients is less intuitive than in linear models (8, 25). These models are rigorously validated using cross-validation techniques or independent external datasets to assess their stability, sensitivity, specificity, and overall diagnostic accuracy. For example, combining features from multiple MRI sequences (e.g., T_1_-weighted, T_2_-weighted, contrast-enhanced images) and integrating peritumoral and intratumoral regions have been shown to improve classification performance (27). Recent advances also include deep learning-based approaches that automatically learn hierarchical features from imaging data. When these are combined with traditional radiomics and clinical features, they can further enhance differentiation between IgG4-ROD and orbital MALT lymphomas. The integration of multimodal radiomics and machine learning thus represents a powerful strategy for noninvasive, accurate diagnosis in orbital lymphoproliferative disorders (8, 27, 32, 33).

#### 2.1.3 Advances in radiomics research for differentiating IgG4-ROD and orbital lymphoma

Recent research leveraging CT and MRI-based radiomics has demonstrated promising diagnostic accuracy in distinguishing IgG4-ROD from orbital lymphomas, particularly MALT lymphoma (8, 25). Studies have reported area under the curve (AUC) values exceeding 0.9 in some models, highlighting the potential of radiomics to surpass the performance of conventional imaging interpretation and even expert radiologists in specific contexts. For instance, fusion models integrating multiparametric MRI radiomics features from both intralesional and perilesional regions have achieved AUCs around 0.91 on external validation, with some studies reporting significant outperformance over subspecialty radiologists whose AUCs ranged between 0.67 and 0.74 (27). Combining radiomics features from different MRI sequences [T_1_WI, T_2_WI, contrast-enhanced T_1_WI (CE-T_1_WI)] further enhances model performance, with multimodal approaches yielding superior classification results compared to unimodal analyses. Moreover, in related orbital differentiation tasks [e.g., ocular adnexal lymphoma (OAL) *vs*. idiopathic orbital inflammation], deep learning models that incorporate multimodal radiomics along with clinical and imaging features have achieved diagnostic AUCs above 0.95, underscoring the value of integrating heterogeneous data sources. Despite these advances, challenges remain, including limited sample sizes in many studies, heterogeneity of imaging protocols, and variability in segmentation methods, which may affect feature reproducibility and model generalizability (30, 34). Additionally, most current studies are retrospective and single-center, highlighting the need for larger, prospective, multicenter trials to validate radiomics models. The potential of multimodal fusion radiomics and advanced machine learning to improve noninvasive diagnosis and reduce unnecessary biopsies in orbital diseases is compelling, but further standardization and external validation are essential before widespread clinical adoption (8, 25, 27, 32, 35). For a detailed summary of the methodologies and diagnostic performance of various AI models in differentiating IgG4-ROD and orbital lymphoma, please refer to **Table 2**.

**Table 2:** Summary of AI model performance in differentiating IgG4-ROD and orbital lymphoma.

Ref. (DOI)	Study focus	AI method/model	Imaging modality	Sample size (IgG4-ROD *vs*. Lymphoma)	Performance (AUC/Accuracy)	Key findings/notes
(26) DOI: 10.1007/s00417-024-06501-1	AI distinguishes IgG4-ROD *vs*. MALT lymphoma (H&E pathology images)	Deep Learning (EVA model)	Histopathology (H&E staining)	97 training/30 testing	AUC: 0.807; Accuracy: 73.3%	Outperformed human ophthalmologists (accuracy 40–60%)
(8) DOI: 10.1155/2021/6668510	MRI radiomics for orbital lymphoma *vs*. IgG4-ROD	Radiomics + Elastic Net	MRI (T1WI, T2WI, CE-T1WI)	78 (42 *vs*. 36)	AUC: 0.727–0.821	Combined CE-T1WI features yielded optimal performance
(25) DOI: 10.1186/s12886-023-03036-7	MRI radiomics for IgG4-ROD *vs*. MALT lymphoma	Radiomics + Support Vector Machine (SVM)	MRI (T1WI, T2WI)	50 (20 *vs*. 30)	AUC: 0.722–0.821	Feature fusion of T1WI and T2WI improved performance
(27) DOI: 10.1186/s12880-025-01771-5	Multi-parametric MRI peritumoral radiomics model	Multi-region Radiomics + LASSO-SVM	Multiparametric MRI (mpMRI)	214 (68 *vs*. 146)	AUC: 0.927 (training), 0.907 (external test)	Significantly outperformed subspecialty radiologists; peritumoral features were highly discriminative

AI, Artificial Intelligence; AUC, Area Under the Curve; CE-T1WI, Contrast-Enhanced T1-weighted image; EVA, which is a vision-centric foundation model used to explore the limits of visual representation; H&E, Hematoxylin and Eosin; IgG4-ROD, Immunoglobulin G4-Related Ophthalmic Disease; LASSO-SVM, Least Absolute Shrinkage and Selection Operator - Support Vector Machine; MRI, Magnetic Resonance Imaging; Orbital MALT Lymphoma, Orbital Mucosa-Associated Lymphoid Tissue Lymphoma; SVM, Support Vector Machine; T1WI, T1-weighted image; T2WI, T2-weighted image.

### 2.2 Deep learning approaches in differentiating IgG4-ROD and orbital MALT lymphoma

#### 2.2.1 Overview of deep learning architectures and mechanisms in orbital imaging

Deep learning, a pivotal subset of AI, has revolutionized medical image analysis by enabling automatic extraction of high-level, abstract features directly from raw imaging data, thereby significantly reducing reliance on handcrafted feature engineering inherent in traditional radiomics (21, 22). In orbital imaging analysis, convolutional neural networks (CNNs) such as residual network (ResNet), Visual Geometry Group (VGG), and Inception architectures have been extensively employed. These architectures excel at capturing local spatial patterns (e.g., edges, textures) and typically offer stable, high-performance results on limited medical datasets. However, standard CNNs often face the “black box” criticism, lacking inherent explainability. To address this, visualization techniques such as Gradient-weighted Class Activation Mapping (Grad-CAM) and saliency maps have been developed. These methods generate heatmaps overlaying the original image, highlighting the specific regions (e.g., the lacrimal gland boundary or peritumoral tissue) that the model focused on to make a prediction, thereby enhancing clinical trust. More recently, Vision Transformers (ViT) have emerged, utilizing self-attention mechanisms to capture long-range dependencies across the entire image. While potentially offering superior performance and intrinsic interpretability through attention maps, Transformers generally require larger datasets to converge compared to CNNs, posing a challenge for rare orbital diseases (36, 37). A common strategy to overcome the limited size of medical imaging datasets is transfer learning, where models pretrained on large-scale natural image datasets (e.g., ImageNet) are fine-tuned on domain-specific orbital imaging data (36). This approach leverages learned general visual features and adapts them to the specific task, enhancing model performance and convergence speed (37). Furthermore, attention mechanisms have been integrated into CNN architectures to enable models to focus selectively on critical regions within orbital images, such as lesions or pathological areas, thereby improving diagnostic accuracy and interpretability. Applications of these deep learning models in orbital imaging span various tasks including image classification for disease differentiation (e.g., distinguishing IgG4-related ophthalmic disease from orbital MALT lymphoma), lesion detection, and segmentation for quantitative assessment of orbital structures (e.g., extraocular muscles and orbital fat). For instance, U-shaped Network (U-Net) and its variants have demonstrated high accuracy in segmenting extraocular muscles and orbital fat on CT and MRI images, facilitating volumetric analyses critical for diseases like thyroid eye disease; for such segmentation tasks, studies have reported Dice coefficients exceeding 0.9, indicating excellent overlap with manual annotations (38, 39). Additionally, CNN-based models have been successfully applied to detect orbital invasion in sinonasal tumors on CT images, with reported diagnostic accuracies surpassing those of non-specialized radiologists and thus enhancing clinical decision-making (37). Similarly, the integration of multimodal imaging features (e.g., T_1_-weighted, T_2_-weighted, contrast-enhanced MRI) with clinical data in deep learning frameworks has further improved differentiation between OAL and idiopathic orbital inflammation, with AUC values reaching above 0.95 (32). These findings from related orbital inflammatory-lymphoproliferative disorders highlight the promising potential of deep learning for the specific task of differentiating IgG4-ROD from orbital MALT lymphoma. These advancements underscore the transformative potential of deep learning architectures, particularly CNNs augmented with transfer learning and attention mechanisms, in enhancing the objectivity, efficiency, and accuracy of orbital disease diagnosis through sophisticated image analysis.

#### 2.2.2 Dataset construction, annotation standardization, and model training optimization

The foundation of successful deep learning applications in differentiating IgG4-ROD from orbital MALT lymphoma lies in the availability of high-quality, well-annotated, and sufficiently large datasets. Precise delineation of orbital lesions and accurate labeling of disease categories are essential, often necessitating consensus annotations by multiple experienced radiologists or pathologists to ensure reliability and reduce inter-observer variability. Given the scarcity of large-scale annotated orbital imaging datasets, multi-center collaboration has become a pivotal strategy to aggregate diverse data, enhancing model generalizability. However, integrating multi-center data introduces challenges such as label inconsistency and heterogeneity in imaging protocols, scanner types, and patient demographics, which can adversely affect model performance (34). To mitigate these issues, standardized annotation protocols and harmonization techniques are employed. Data augmentation methods, including geometric transformations (rotation, flipping, cropping), intensity variations (brightness, contrast adjustments), and synthetic data generation via generative adversarial networks (GANs), have been utilized to artificially expand dataset size and diversity, thereby reducing overfitting and improving robustness (40). GANs, in particular, have shown promise in generating realistic synthetic orbital images that preserve pathological features critical for training. Model training optimization involves careful selection of loss functions, potentially tailored for imbalanced data or segmentation tasks (e.g., Dice loss or focal loss, which emphasize difficult-to-classify samples). Optimizers like Adam or stochastic gradient descent with momentum, combined with learning rate scheduling and regularization techniques (dropout, weight decay), are employed to enhance convergence and prevent overfitting. Recent studies have also explored advanced training strategies, including local critic training and bypass algorithms, to improve computational efficiency and model stability (41, 42). Additionally, privacy-preserving distributed learning frameworks, such as federated learning and split learning, enable collaborative model training across institutions without sharing raw data, addressing data privacy concerns while leveraging multi-center datasets (34, 43). Collectively, these strategies in dataset construction, annotation standardization, and training optimization are critical to developing robust, accurate, and clinically applicable deep learning models for orbital disease differentiation.

#### 2.2.3 Performance evaluation of deep learning models and clinical translation

A comprehensive overview of the current research landscape, including the AI approaches, data characteristics, and reported diagnostic outcomes for differentiating IgG4-ROD and orbital MALT lymphoma, as well as related orbital lymphoproliferative disorders, is provided in **Table 2**. Evaluating the diagnostic performance of deep learning models in differentiating IgG4-ROD from orbital MALT lymphoma involves comprehensive assessment metrics such as sensitivity, specificity, accuracy, and the AUC value (30). In related orbital differentiation tasks (e.g., OAL *vs*. idiopathic orbital inflammation), multiple studies have demonstrated that models integrating multimodal imaging features and clinical data achieve superior performance, with AUC values often exceeding 0.9, indicating high discriminative capability (32, 33). These results suggest a strong potential for similar high performance in differentiating IgG4-ROD from orbital MALT lymphoma. Comparative analyses reveal that fused intra- and peritumoral feature models outperform those relying solely on intratumoral features, highlighting the importance of contextual information in orbital lesion characterization (33). Despite promising quantitative results, several challenges impede clinical translation. A major obstacle is the limited interpretability of deep learning models, often perceived as “black boxes,” which hinders clinician trust and acceptance. Efforts to enhance explainability through attention maps, saliency visualization, and feature importance analyses are ongoing but require further refinement. Data privacy and regulatory compliance also pose significant hurdles, necessitating secure model deployment frameworks and adherence to healthcare data protection standards (43). Moreover, variability in imaging protocols and patient populations across institutions can affect model generalizability, underscoring the need for extensive external validation and prospective clinical trials. Looking forward, integrating radiomics with deep learning in a multimodal fusion approach holds promise for improving diagnostic accuracy and robustness. Combining imaging data with genomic, proteomic, and clinical information through advanced machine learning frameworks may enable personalized diagnostic and therapeutic strategies. Additionally, the development of user-friendly software tools and incorporation of deep learning models into existing clinical workflows will facilitate adoption. Ultimately, the synergy between radiomics and deep learning, coupled with multidisciplinary collaboration, is anticipated to transform orbital disease diagnosis by providing robust decision support for clinicians, thereby enhancing precision medicine and patient outcomes (33, 44). It is crucial to emphasize that these AI tools are envisioned as powerful adjuncts to clinical judgment and traditional diagnostic pathways, rather than standalone replacements for definitive histopathological biopsy.

## 3 Discussion

In conclusion, the integration of AI technologies—particularly radiomics and deep learning—into the diagnostic landscape of IgG4-ROD versus orbital MALT lymphoma represents a transformative advancement with substantial clinical promise. From an expert perspective, these cutting-edge methodologies offer a potential paradigm shift by enabling non-invasive, objective, and high-precision imaging analyses that can significantly enhance differential diagnosis accuracy (21, 24), serving as crucial decision-support tools to guide clinical judgment and optimize patient management, rather than replacing the definitive role of histopathological biopsy. This approach aims to support more informed and timely clinical decision-making and potentially reduce the need for invasive procedures.

A critical synthesis of the reviewed literature reveals distinct methodological profiles for these two approaches. Radiomics excels in interpretability and data efficiency; by quantifying biologically relevant features (e.g., texture heterogeneity reflecting storiform fibrosis), it offers transparent insights into the underlying pathology and performs relatively well even with smaller datasets typical of rare orbital diseases (8, 25). However, its dependence on precise, often manual, segmentation and handcrafted feature engineering introduces potential for inter-observer variability and limits scalability. In contrast, deep learning offers a streamlined, end-to-end workflow with superior automated feature extraction capabilities, reducing human bias and potentially achieving higher diagnostic accuracy by capturing abstract, non-linear patterns (26, 32). Yet, its clinical application is currently constrained by the “black box” nature of decision-making and a significant requirement for large, annotated datasets to prevent overfitting. Therefore, the evidence suggests a synergistic path forward: integrating radiomics’ explicit, interpretable features with deep learning’s powerful abstract representations—alongside clinical data—can mitigate individual limitations, fostering robust diagnostic models that are both high-performing and clinically trustworthy (21, 27, 32).

Nevertheless, despite these promising developments, the field remains in an early exploratory phase with several critical challenges impeding the transition from research to routine clinical practice. Predominantly, existing studies suffer from limited sample sizes and are often confined to single-center, retrospective designs, which constrain the generalizability and robustness of AI models (25). The heterogeneity of imaging data across studies, compounded by a lack of standardized acquisition protocols and lesion annotation criteria, further undermines model reproducibility and cross-institutional applicability. Moreover, the intrinsic “black box” nature of many deep learning algorithms poses significant barriers to clinical acceptance, as insufficient model interpretability diminishes clinician trust and complicates regulatory approval processes (30, 34).

Addressing these multifaceted challenges requires a concerted, multidisciplinary effort. Future research must prioritize the establishment of large-scale, multicenter, prospective cohorts to generate high-quality, standardized datasets that better capture the diversity of patient populations and imaging characteristics. Integrative approaches that fuse multimodal imaging data—such as CT, MRI, and potentially Positron Emission Tomography/Computed Tomography (PET/CT)—with clinical, pathological, and genomic information hold promise for constructing comprehensive diagnostic frameworks that reflect the complex biology of these diseases. Furthermore, advancing explainable AI (XAI) techniques is imperative to enhance model transparency, enabling clinicians to understand and validate AI-driven predictions, thereby fostering greater confidence and facilitating clinical integration (30, 32, 38, 39). Rigorous clinical validation studies, alongside cost-effectiveness analyses, are essential to demonstrate tangible benefits in patient outcomes and healthcare resource utilization.

In essence, while AI-driven radiomics and deep learning have already demonstrated remarkable potential in differentiating IgG4-ROD from orbital MALT lymphoma, their full clinical impact hinges on rigorously addressing current methodological and translational hurdles, particularly the pervasive issues of limited sample sizes, retrospective single-center study designs, and the critical need for comprehensive external validation. By harmonizing diverse research perspectives and fostering collaborative innovation, the field can move toward realizing AI as a vital adjunct in the diagnostic armamentarium for orbital diseases. Ultimately, this will translate into improved diagnostic accuracy, personalized treatment strategies, and enhanced prognostic outcomes for patients afflicted with these challenging ophthalmic conditions.
